# Comparing Resident Team Performance in Complex Surgical Oncology: A Single-Institution Cohort Study

**DOI:** 10.1245/s10434-026-19976-5

**Published:** 2026-06-14

**Authors:** Rachael C. Acker, James E. Sharpe, Shane Williams, Sarah I. Landau, James Rowe, Douglas Fraker, Giorgos C. Karakousis, Heather Wachtel, Rachel R. Kelz

**Affiliations:** 1 Department of Surgery, University of Pennsylvania Health System, Philadelphia, PA USA; 2 Center for Surgery and Health Economics, Philadelphia, PA USA; 3 Leonard Davis Institute of Health Economics, University of Pennsylvania, Philadelphia, PA USA

## Abstract

**Background:**

Team dynamics influence team performance and patient outcomes in surgery, yet data on resident-led teams are scarce. This study aimed to compare patient outcomes across resident-led teams in complex surgical oncology. We hypothesized that patient outcomes would vary by team assignment.

**Methods:**

This was a retrospective cohort study of resident-led teams who contributed more than 10 surgical oncology operations (colectomy, hepatectomy, pancreatectomy, or thyroidectomy) to the National Surgical Quality Improvement Project registry at a single university-based hospital (2018–2025). The primary outcome was presence of any adverse event, including mortality and postoperative complications. Length of stay and 30-day readmissions were also examined. Mixed-effects regression estimated expected outcome probabilities for each patient. For each team, observed-minus-expected (O-E) outcome rates were calculated to assess performance.

**Results:**

In total, 145 teams cared for a median of 22 patients (interquartile interval 16– 27; *n* = 2919). Five teams demonstrated poor performance based on risk-adjusted adverse event rates (O-E rates: 6.9% and 22.3%). A total of 13 teams had significantly longer risk-adjusted length of stays than expected (O-E between 0.4 and 3.5 days), and seven teams had shorter risk-adjusted length of stays than expected (O-E between -1.1 and -0.7 days). Three teams had higher risk-adjusted readmission rates than expected (O-E between 4.9% and 6.8%). Two teams performed poorly across all three outcomes.

**Conclusions:**

Variation in team performance can be measured using valid and reliable risk-adjusted patient outcomes in complex surgical oncology. This may provide meaningful feedback with benchmarking to identify teams that require more supervision.

**Supplementary Information:**

The online version contains supplementary material available at 10.1245/s10434-026-19976-5.

Resident physicians in the USA provide care to an estimated 18 million patients across 1000 teaching hospitals.^1^ In most academic medical centers, surgical teams are led by residents. The patient outcomes associated with resident involvement in surgical care are variable.^2–7^ For example, one study demonstrated that greater resident autonomy in the operating room was associated with higher complication rates.^7^ Conversely, another study in surgical oncology demonstrated improved outcomes, with lower mortality and failure-to-rescue rates when residents were involved in care.^2^ Although advanced practice provider support may exist to provide continuity across teams, overnight and weekend coverage at academic institutions is most often provided exclusively by residents.

Across multiple specialties, positive team dynamics are associated with low adverse event rates and high patient satisfaction.^8,9^ Teams that promote psychological safety and communication demonstrate better performance and exhibit more information sharing in the operating room.^10,11^ These practice patterns are learned during residency, and multiple studies have shown an association between professionalism and communication scores during training and patient complaints and complication rates after the transition to practice.^12–18^ Despite its critical importance to patient care, little feedback exists on team performance during surgical training, and many new physicians struggle to lead multi-disciplinary teams after the transition to practice.^19–21^

The Quality In Training Initiative (QITI) links National Surgical Quality Improvement Project (NSQIP) procedures to the resident teams who perform the operations and care for patients postoperatively.^22^ However, to date, many hospital sites do not participate in QITI, and it is challenging to interpret resident performance without risk adjustment of these data.^23^ Therefore, this exploratory study aimed to compare risk-adjusted patient outcomes of resident teams in complex surgical oncology as a mechanism for feedback with benchmarking during training and targeted improvement. We predicted that measurable differences in performance would exist across resident teams.

## Methods

### Data Sources and Population

This was a retrospective cohort study of general surgery resident teams who contributed more than 10 surgical oncology operations (colectomy, hepatectomy, pancreatectomy, or thyroidectomy) to the NSQIP and QITI registries at a single university-based hospital from January 1, 2018, to June 30, 2025.^22,24^ These four operations were chosen because they represent targeted cases within the NSQIP database.^25^ The NSQIP and QITI databases were linked with patient identifiers such that patients and operations were assigned an attending surgeon, operative resident, and resident team. The QITI database captures the service that cared for each patient postoperatively, the month, and the year of the operation. Resident teams were defined by the combination of service (surgical oncology or colorectal surgery), month, and year in which the operation was performed. Resident rotation assignments change monthly at our institution. Thus, the monthly team definition captures the unique combination of residents caring for each patient. Patients were assigned to the resident team of the operative resident. This is consistent with other surgical outcomes research assignment strategies for team-based care scenarios. For example, in emergency general surgery, patient outcomes are most often attributed to the operative surgeon, even when that surgeon did not admit the patient or may have been on call only.^26,27^

Low-volume teams who contributed fewer than 10 operations were excluded. Patients were excluded if they underwent an operation for benign disease or were operated on by an attending surgeon who contributed fewer than 10 cases to the database (Supplement 1).

### Exposure and Outcomes

The primary exposure was the anonymized resident team. The primary outcome was a composite metric of any adverse event, including 30-day mortality, reoperation, readmission, and postoperative complications. Secondary outcomes included length of stay (LOS) and individual adverse event rates.

### Covariates

Covariates included operation (hepatectomy, colectomy, pancreatectomy, thyroidectomy), acuity (emergent vs. elective), surgical approach (open vs. minimally invasive including laparoscopic or robotic approaches), and post-graduate year of the operative resident. Operations were classified based on Current Procedural Terminology codes (Supplement 2). Indications for operations were classified into three categories—malignancy, suspected malignancy, or unknown at the time of the operation. Patient age, sex, American Society of Anesthesiologists (ASA) class (<3 or ≥3), comorbidity sum, presence of disseminated cancer, immunosuppression, and albumin level (≤3 or >3 g/dL) were extracted. The ASA and albumin cutoffs were selected based on previous studies using the NSQIP database.^28,29^

### Analytic Strategy

Univariate analyses compared operation and patient characteristics across resident teams using Kruskal–Wallis and Fisher’s exact tests. Mixed-effects regression models were used to estimate the expected outcome probabilities for each patient, controlling for potential confounders as fixed effects and attending surgeon identifier as a random effect. For each team, observed minus expected (O-E) outcome rates were then calculated with 95% confidence intervals to assess performance. Rates <0 were considered superior performance, and rates >0 were considered inferior performance. Confidence intervals that excluded 0 were considered outliers.

A stratified analysis was performed to examine teams that were performance outliers across all three metrics compared with other teams. Patient and operative characteristics as well as rates of adverse events and failure to rescue (defined as mortality after a complication^30^) were examined using Fisher’s exact tests. Based on the low mortality rate overall, failure to rescue was not examined as a primary outcome.

Given the short LOS and the low frequency of adverse events after thyroidectomy, a subgroup analysis was performed on only patients who underwent hepatectomy, colectomy, or pancreatectomy operations.

Analyses were completed in R 4.4.1 (R Core Team, Austria, Vienna), Stata 17 (StataCorp LLC, College Station, TX, USA), and SAS/STAT 9.4 (SAS Institute Inc., Cary, NC, USA). Two-sided *p*-values of ≤0.05 were considered statistically significant. This study was exempted from review by the University of Pennsylvania institutional review board (protocol 859104).

## Results

A total of 145 resident teams cared for 2919 postoperative patients with a median of 22 patients (interquartile interval [IQI] 16–27) per team. A total of 12 attending surgeons were represented (Supplement 1). A majority of operations were performed for biopsy-proven malignancies (a median of 50% of operations per team [IQI 24–73]), and a median 26% of each team’s cases (IQI 10–41) were performed for suspected malignancies (Table 1).

**Table 1:** Patient characteristics by team

Characteristics	Total patients *n(%)*	Median number of patients per team [IQI] *n=142 teams*	*P*-value
All patients	2,919	22 (16–27)	
Operation			
Hepatectomy	344 (12)	2 (0–4)	<0.001
Pancreatectomy^a^	264 (9)	1 (0–2)	<0.001
Colectomy^a^	746 (26)	2 (0–11)	<0.001
Thyroidectomy^a^	1565 (54)	3 (0–22)	<0.001

	Total patients *n(%)*	Median proportion of patients treated per team % [IQI]	
*Minimally invasive, yes*	526 (18)	50 (0–73)	< 0.001
Indication for operation			
Cancer	1166 (40)	50 (24–73)	< 0.001
Suspicion of cancer	849 (29)	26 (10–41)	< 0.001
Unknown	904 (31)	27 (10–39)	< 0.001
Emergent case, yes	8 (<1)	0 (0–0)	0.05
Age, years	58 (45, 68)	57 (54–62)	<0.001
Female, yes	1847 (63)	60 (50–71)	<0.001
Race			
White	2171 (74)	74 (68–83)	0.01
Black	417 (14)	13 (8–20)	0.02
Asian	146 (5)	4 (0–8)	0.95
Other	185 (6)	6 (0–9)	0.51
Primary payer			
Medicare	919 (31)	32 (24–45)	<0.001
Medicaid	233 (8)	7 (0–11)	0.11
Private	1741 (60)	58 (46–68)	<0.001
Self-Pay	7 (<1)	0 (0–0)	0.83
Other	19 (1)	0 (0–0)	0.71
Sum of NSQIP comorbidities	1 (0–1)	0.8 (0.6–0.9)	0.003
ASA class ≥3	1,564	57 (46–68)	<0.001
Comorbidities (list)			
Acute kidney injury, yes	1 (<1)	0 (0–0)	0.17
Ascites, yes	4 (<1)	0 (0–0)	0.05
Bleeding disorder, yes	37 (1)	0 (0–0)	0.04
COPD, yes	45 (2)	0 (0–2)	0.001
Diabetes, yes	435 (15)	15 (9–20)	0.51
Dialysis, yes	16 (1)	0 (0–0)	0.31
Disseminated cancer, yes	303 (10)	9 (0–15)	<0.001
Functional health status, dependent	7 (<1)	0 (0–0)	0.03
Heart failure, yes	59 (2)	0 (0–4)	0.02
Hypertension, yes	1143 (39)	40 (31–50)	0.05
Immunosuppressive therapy, yes	145 (5)	5 (0–8)	0.01
Sepsis, yes	6 (<1)	0 (0–0)	0.04
Body mass index	28 (24–33)	29 (27–30)	0.05
Albumin ≤3 g/dL	14 (<1)	0 (0–0)	0.72

Data are presented as n (%) or median (interquartile interval) unless otherwise indicated. *P*-values measure variation across teams using Kruskal–Wallis and Fisher’s exact tests. ASA American Society of Anesthesiologists, COPD Chronic obstructive pulmonary disorder, NSQIP National surgical quality improvement project. ^a^See Supplement 3 for extent of operation performed (total vs. partial)

The median patient age treated by each team was 57 years (IQI 54–62). The proportion of patients who received immunosuppressive therapy, with ASA class ≥3, and disseminated cancer differed across teams (*p *< 0.001) (Table 1). The proportion of patients with ascites and albumin ≤3 g/dL was similar across teams (*p* = 0.05 and *p* = 0.72).

### Unadjusted Outcomes

A total of 433 patients (15%) had at least one adverse event. There were significant differences in rates across teams, with a median rate of 14% (IQI 9–23) per team (*p* <0.001; Table 2). The median LOS was 4 days (IQI 2–6) per team (*p* <0.001). The median 30-day readmission rate was 6% (IQI 0–10) per team (*p* = 0.003). Wound complications were the most frequent postoperative complications, affecting 163 patients (6%) with a median rate of 5% (IQI 0–10) per team (*p *< 0.001).

**Table 2:** Unadjusted outcomes by team

Postoperative complication	Total patients *n=2919*	Median rate per team (%) *n=145 teams*	*P*-value
Any adverse event, yes	433 (15)	14 (9–23)	< 0.001
30-day readmission	181(6)	6 (0–10)	0.003
30-day return to OR	54 (2)	0 (0–3)	0.71
Mortality	4 (<1)	0 (0–0)	0.07
Wound complications	163 (6)	5 (0–10)	< 0.001
Respiratory complications	57 (2)	0 (0–3)	0.08
CNS complications	5 (<1)	0 (0–0)	0.96
Cardiac complications	10 (<1)	0 (0–0)	0.15
Urinary complications	65 (2)	0 (0–5)	< 0.001
Other complications	189 (6)	6 (0–10)	< 0.001
Length of stay, days	1 (1–5)	4 (2–6)	< 0.001

Data are presented as n (%) or median (interquartile interval) unless otherwise indicated. *P*-values measure variation across teams using Kruskal–Wallis and Fisher’s exact tests

### Adjusted Outcomes

**Fig. 1:**
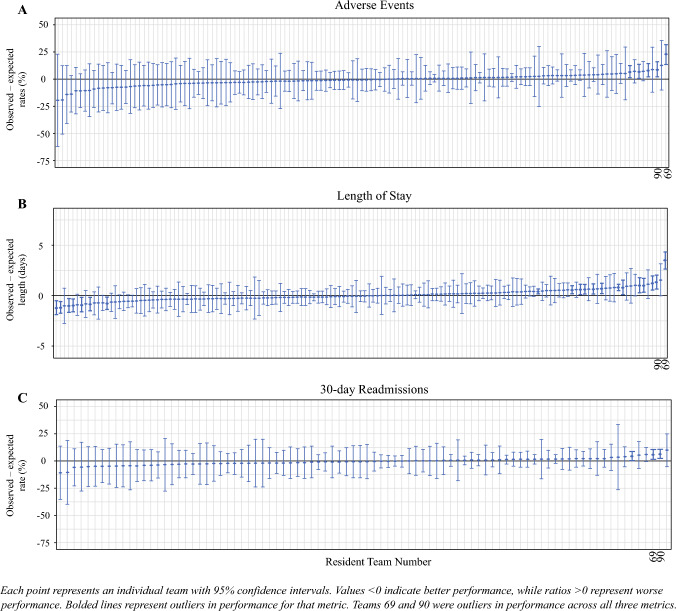
Caterpillar plots depict observed minus expected rates of adverse events (Panel **A**), length of stay (Panel **B**), and 30-day readmission (Panel **C**) by individual team

After adjustment for potential confounders including attending surgeon, operative, and patient characteristics, five teams were poor performance outliers in adverse events, with O-E rates between 6.9% and 22.3% (Fig. 1). Thirteen teams had significantly longer adjusted LOS than expected (O-E between 0.4 and 3.5 days), and seven teams had shorter adjusted LOS than expected (O-E between − 1.1 and − 0.7 days). Three teams were poor performance outliers in 30-day readmissions (O-E between 4.9 and 6.8%).

Of the 71 teams that performed at least 10 hepatectomies, colectomies, and pancreatectomies (excluding thyroidectomies), one team was an exceptional performance outlier in 30-day readmissions (O-E rate: -28.5%) (Supplement 4). There were differences across the other teams, with O-E rates ranging from -28.5% to +10.1%, but these did not reach statistical significance. A wide range in adverse event rates (O-E ranging from -19.6% to +12.4%) and LOS (O-E values ranging from − 1.0 day to +1.5 days) was observed across teams without statistically significant differences.

### Performance Outliers

Two teams (#69 and #90) performed poorly across all three outcomes. These teams had adverse event rates of 43% (*n* = 7/16) and 20% (*n* = 4/20). The mean adverse event rate of the other 143 teams was 17%. The failure-to-rescue rate was 9% (*n* = 1/11) for the two poorly performing teams and 0.7% (*n* = 3/422) for the other 143 teams (*p* = 0.09).

These two outlier teams had higher rates of wound complications than the other 143 teams (17% vs. 6%, *p* = 0.02). They also had higher rates of urinary (8% vs. 3%, *p* = 0.05) and respiratory (6% vs. 2%, *p* = 0.16) complications than the other 143 teams, but these did not reach statistical significance. Based on the occurrences, we compared patients on risk factors for these events and found no differences in the proportion of patients with ASA ≥3 or with any of the individual comorbidities examined treated by these two teams compared with the other 143 teams (all *p* >0.05).

## Discussion

This study demonstrated the feasibility of measuring resident-led team performance using valid and reliable risk-adjusted patient outcomes. Using this method, we identified variation in adverse event rates, LOS, and 30-day readmission rates across teams. LOS was particularly sensitive to team performance, with the greatest number of teams performing differently than expected. Few teams demonstrated higher, and no teams demonstrated lower, rates of adverse events or 30-day readmissions than expected. Two teams performed poorly across all three metrics.

Our findings indicate that some metrics may be more attributable to team performance than others. The greatest number of teams in our study were performance outliers with respect to LOS. This may be due to the high degree of autonomy that residents have in the postoperative care of patients, and this aligns with previously published studies that demonstrate that residents impact LOS in surgery and emergency medicine.^31–34^ These differences may be particularly pronounced over the weekend when staffing is reduced or when transitions in care occur. For example, one study of over 95,000 patients showed decreased discharged rates and longer LOS on days when residents change rotations.^35^ Further, a previously published study demonstrated differences in hospital-based failure-to-rescue rates between patients cared for with and without resident involvement.^2^ Although mortality was a rare event, the two teams that performed poorly across all three outcomes had higher failure-to-rescue rates than the other teams, suggesting that failure to rescue may be an important metric of resident team performance in a local context. Without thyroidectomy cases, there were clinically meaningful differences in adverse event rates and LOS across teams. However, the confidence intervals were wide, likely because of the smaller number of teams and number of cases per team included in the analysis, and these differences did not reach statistical significance.

Multiple studies have demonstrated the link between team dynamics and patient outcomes.^8–11^ Although the cause of the variation observed across teams in this study is not known, both the Accreditation Council for Graduate Medical Education Milestones and the new American Board of Surgery Entrustable Professional Activities identify the ability to lead, work in, and collaborate with interdisciplinary teams and communicate with patients and other healthcare providers as essential behaviors of a practicing surgeon.^36,37^ However, the definitions and criteria for evaluating these skills are not explicit.^38,39^ The use of real-world patient data to understand team performance may provide objective adjuncts to our current assessments that are tied to essential outcomes. As medical education requires a challenging balance between the provision of resident autonomy to promote learning and ensuring patient safety, the NSQIP outcomes data may also help attending surgeons titrate their supervision of teams based on a senior resident’s track record of performance as a team leader.

A majority of the teams in this study exhibited similar performance. This may reflect the role of nursing staff, advanced practice providers, and postoperative protocols in standardizing care. Alternatively, these findings may reflect consistency in the patient care that most resident teams provide and the rarity of performance outliers. Therefore, teams who fail to meet a minimum threshold of competency likely require intervention. The residents leading those teams may also benefit from remediation. Conversely, teams exhibiting exceptional performance may provide critical insights for program directors to understand the team dynamics that lead to positive outcomes.

Ultimately, feedback with benchmarking is critical to professional growth and skill development and has been shown to lead to improved performance among attending surgeons.^40–50^ Most residents do not currently have access to real-world performance data.^52^ Exposing residents, especially senior residents leading teams, to their patient outcomes—the data by which they will be assessed as attending surgeons—may help them integrate this type of feedback into their own reflective practice earlier in their careers.

This study had several limitations. These outcomes are not the sole measure of team performance and must be integrated with other assessments of performance. However, these outcomes do show significant variation across resident teams and provide a powerful method for program directors to flag teams that require additional observation. The NSQIP database contains only a random sample of operations. More performance differences may exist and could be measured with the availability of more comprehensive case data for each team. However, given the random sampling and similarities in patient characteristics noted in this study, this sample should be representative of each team’s case mix. Multiple teams may have been involved in caring for each patient given that residents change rotations each month. We performed an intention-to-treat analysis to measure the effectiveness of team performance on patient outcomes. Future studies could address the issue of exposure time to more accurately assess efficacy. Team dynamics, including the leadership style of the chief resident, may affect the patient outcomes observed in this study and are not reflected in these data. Many institutions have dedicated leadership training curricula, but there were no such efforts at this program during the study period. We examined only team performance rather than individual performance in this study. The individual operative resident likely also contributes to patient outcomes and requires future investigation. The degree of resident autonomy in the operating room and on the wards is not known and may influence the patient outcomes observed. Therefore, these data should ultimately be used and interpreted in conjunction with other assessment data rather than as isolated assessment metrics.

## Conclusions

Risk-adjusted patient outcomes can be used to measure variation in resident team performance in complex surgical oncology. The greatest variation across teams was observed in LOS, arguably the study outcome that residents may influence the most. These methods may provide residents with important feedback and may flag outlier teams that could benefit from additional observation. Incorporating these data into surgical training could also expose residents to feedback with benchmarking earlier in their careers to normalize the range of performance and promote practices of continuous learning and improvement.

## Supplementary Information

Below is the link to the electronic supplementary material.

Supplementary file1 (DOCX 621 kb)

Supplementary file2 (DOCX 33 kb)
