# Supplementary material for: Comparing Resident Team Performance in Complex Surgical Oncology: A Single-Institution Cohort Study
Source: Ann Surg Oncol. 2026 Jun 14;33(9):8282–9. doi: 10.1245/s10434-026-19976-5 (PMC13452783; doi:10.1245/s10434-026-19976-5)
Supplement: Supplementary file 1 [file 10434_2026_19976_MOESM1_ESM.docx]

**Supplement 1.** Cohort diagram.

**Supplement 2.** Classification of extent of operation (i.e., partial vs. complete/total operations) by Current Procedural Terminology (CPT) code.

| **CPT Code** | **Operative Extent** | **Description** |
| --- | --- | --- |
| **Pancreatectomy** | | |
| 48120 | **Partial** | Excision of lesion of pancreas (e.g., cyst, adenoma) |
| 48140 |  | Distal subtotal pancreatectomy, w/ or w/o splenectomy; without pancreatojejunostomy |
| 48145 |  | Distal subtotal pancreatectomy, w/ or w/o splenectomy; with pancreatojejunostomy |
| 48148 |  | Excision of ampulla of Vater (pancreas related) |
| 48150 |  | Pancreatectomy, proximal subtotal with total duodenectomy, partial gastrectomy, choledochoenterostomy & gastrojejunostomy; with pancreatojejunostomy |
| 48152 |  | Pancreatectomy, proximal subtotal with total duodenectomy, partial gastrectomy, choledochoenterostomy & gastrojejunostomy; without pancreatojejunostomy |
| 48153 |  | Pancreatectomy, proximal subtotal with near-total duodenectomy, choledochoenterostomy & duodenojejunostomy (pylorus-sparing Whipple) with pancreatojejunostomy |
| 48154 |  | Pancreatectomy, proximal subtotal with near-total duodenectomy, choledochoenterostomy & duodenojejunostomy (pylorus-sparing Whipple) without pancreatojejunostomy |
| 48155 | **Total** | Pancreatectomy, total |
| 48146 |  | Pancreatectomy, distal “near-total” with preservation of duodenum |
| **Colectomy** | | |
| 44140 | **Partial** | Colectomy, partial; with anastomosis |
| 44141 |  | Colectomy, partial; with skin-level cecostomy or colostomy |
| 44143 |  | Colectomy, partial; with end colostomy and closure of distal segment |
| 44144 |  | Colectomy, partial; with resection, with colostomy or ileostomy and creation of mucofistula |
| 44145 |  | Colectomy, partial; with coloproctostomy (low pelvic anastomosis) |
| 44146 |  | Colectomy, partial; with coloproctostomy (low pelvic anastomosis) with colostomy |
| 44147 |  | Colectomy, partial; abdominal and transanal approach |
| 44160 |  | Colectomy, partial; with removal of terminal ileum with ileocolostomy |
| 44204 |  | Laparoscopy, surgical; colectomy, partial, with anastomosis |
| 44205 |  | Laparoscopy, surgical; colectomy, partial, with removal of terminal ileum with ileocolostomy |
| 44206 |  | Laparoscopy, surgical; colectomy, partial, with end colostomy & closure of distal segment |
| 44207 |  | Laparoscopy, surgical; colectomy, partial, with anastomosis + coloproctostomy (low pelvic) |
| 44208 |  | Laparoscopy, surgical; colectomy, partial, with anastomosis + colostomy (low pelvic) |
| 44150 | **Total** | Colectomy, total; abdominal, without proctectomy; with ileostomy or ileoproctostomy |
| 44151 |  | Colectomy, total; abdominal, without proctectomy; with continent ileostomy |
| 44155 |  | Colectomy, total; abdominal, with proctectomy; with ileostomy |
| 44156 |  | Colectomy, total; abdominal, with proctectomy; with continent ileostomy |
| 44157 |  | Colectomy, total; abdominal, without proctectomy; with ileoanal anastomosis, includes loop ileostomy & rectal mucosectomy when performed |
| 44158 |  | Colectomy, total; abdominal, without proctectomy; with ileoanal anastomosis, creation of ileal reservoir (S or J), includes loop ileostomy & rectal mucosectomy when performed |
| 44210 |  | Laparoscopy, surgical; colectomy, total, abdominal, without proctectomy; with ileostomy or ileoproctostomy |
| **Hepatectomy** | | |
| 47120 |  | Hepatectomy, resection of liver; partial lobectomy |
| 47122 |  | Hepatectomy, resection of liver; trisegmentectomy |
| 47125 |  | Hepatectomy, resection of liver; total left lobectomy |
| 47130 |  | Hepatectomy, resection of liver; total right lobectomy |
| **Thyroidectomy** | | |
| 60200 | **Partial** | Excision of cyst or adenoma, thyroid |
| 60210 |  | Partial thyroid lobectomy, unilateral ± isthmusectomy |
| 60212 |  | Partial thyroid lobectomy, unilateral with contralateral subtotal lobectomy |
| 60220 |  | Thyroid lobectomy, unilateral ± isthmusectomy |
| 60225 |  | Thyroid lobectomy, unilateral with contralateral subtotal lobectomy |
| 60260 |  | Removal of remaining thyroid tissue after prior partial thyroidectomy (completion) |
| 60240 | **Total** | Thyroidectomy, total or complete |
| 60254 |  | Thyroidectomy, total or subtotal for malignancy; with radical neck dissection |
| 60270 |  | Thyroidectomy, including substernal thyroid; sternal split/transthoracic approach |
| 60271 |  | Thyroidectomy, including substernal thyroid; cervical approach |
| 60252 |  | Thyroidectomy, total or subtotal for malignancy; with limited neck dissection |
| Patients were identified for inclusion based on their primary procedure codes. Thyroidectomy patients were classified as undergoing a lateral neck dissection if they either had CPT code 60252 or had the presence of a lateral neck dissection code as a secondary code in addition to their thyroidectomy. | | |

**Supplement 3.** Extent of operation performed by resident team.

| **Characteristics** | **Total Patients**  n (%) | **Median number per team** [IQI]  *n=142 teams* | **P-value** |
| --- | --- | --- | --- |
| **Pancreatectomy** *n=264 patients* |  |  |  |
| Total | 2 (<1) | 0 [0, 0] | 0.01 |
| Partial | 262 (99) | 1 [0, 2] | <0.001 |
| **Colectomy** *n=746 patients* |  |  |  |
| Total | 22 (3) | 0 [0, 0] | 0.001 |
| Subtotal | 724 (97) | 2 [0, 11] | <0.001 |
| **Thyroidectomy** *n=1565 patients* |  |  |  |
| Total thyroidectomy | 1255 (80) | 3 [0, 17] | <0.001 |
| Partial thyroidectomy | 310 (20) | 0 [0, 4] | <0.001 |
| Lateral neck dissection | 18 (1) | 0 [0, 0] | 0.89 |
| *P-values measure variation across teams using Kruskal-Wallis tests. IQI= Interquartile interval.* | | | |

**Supplement 4.** Subgroup analysis: caterpillar plots of observed minus expected values by individual team excluding thyroidectomy cases.
